# Infection control practices among EMS providers in prehospital settings: a scoping review of compliance and barriers

**DOI:** 10.1017/ash.2025.10194

**Published:** 2025-10-17

**Authors:** Abdulrahman N. Alsaleem, James V. Lawler, Kimberly A. Harp

**Affiliations:** 1 College of Graduate Studies, University of Nebraska Medical Center, Omaha, NE, USA; 2 College of Applied Medical Sciences, King Saud bin Abdulaziz University for Health Sciences, Al Ahsa, Saudi Arabia; 3 Department of Internal Medicine, College of Medicine, University of Nebraska Medical Center, Omaha, NE, USA; 4 Leon S McGoogan Health Sciences Library, University of Nebraska Medical Center, Omaha, NE, USA

## Abstract

**Objectives:**

Infection control practices are essential in any healthcare setting, including the prehospital setting, to protect both patients and healthcare providers. Yet, many unknowns exist about these practices in EMS settings. This review explores infection control practices in emergency medical services by exploring compliance rates and barriers documented in the published literature.

**Methods:**

To identify relevant articles, a comprehensive search of the databases Embase, PubMed, and CINAHL was conducted. Furthermore, a hand-search of the reference lists from the included studies was performed to identifyadditional relevant research. The review followed the Arksey and O’Malley framework and was reported in accordance with PRISMA-ScR guidelines.

**Results:**

A total of 184 records were initially identified—159 through database searches and 25 through hand-searching the reference lists. After removing duplicates and excluding non-English records and those published outside the specified time frame, 162 records remained. Title and abstract screening narrowed these down to 52 articles for full-text review, of which 16 met the inclusion criteria and were included in the final review.

**Conclusions:**

The findings indicate that compliance rate with infection control practices among EMS providers falls short of the desired levels. However, most of the included studies utilized self-reported methods to assess compliance, which may not accurately reflect actual practices. Therefore, future research should incorporate direct observations to gain a clearer understanding of current practices. Furthermore, this review identified that the barriers faced by EMS providers are complex and multifaceted, necessitating a comprehensive strategy to effectively address these challenges.

## Introduction

Emergency Medical Services (EMS) providers play a fundamental role in the healthcare system, often being the first point of contact for patient encounters with healthcare. EMS transports approximately 28 million patients to hospitals annually in the United States.^1^ EMS providers regularly work under challenging conditions that increase their exposure to many occupational risks, including infectious diseases.^2,3^ Failure to comply with infection control guidelines can have profound consequences. While hospital infection prevention and control (IPC) is now a focus for quality improvement in healthcare, research on IPC in the EMS context historically has been limited.^4,5^ The existing literature on this topic heavily focuses on bioterrorism and disaster preparedness rather than exploring basic infection control practices in prehospital settings.^6^

A lack of standard definitions for healthcare-associated infections (HAIs) in the prehospital setting highlights the aforementioned gaps. Because this review focuses on EMS, we will use “medical transport–associated infection” (MTAI). MTAI is a subset of HAIs and refers to “any infection acquired as a direct effect of exposure in a medical transport setting”.^7^ Studies that assess the association between patient transport by EMS providers and MTAI rates are few, but they show that patients may be at a higher risk of infection if transported by ambulance. For example, a 2021 study found that patients arriving at the emergency department by ambulance were almost four times more likely to develop methicillin-resistant Staphylococcus aureus or vancomycin-resistant Enterococcus (VRE) within 30 days of transport compared to those arriving in a private vehicle.^8^ Research indicates that infection rates among EMS providers can be up to three times higher than the general population.^9^ Clearly, adherence to infection control practices is vital for protecting both EMS providers and patients.

Studies assessing IPC compliance among EMS providers report a wide range of compliance rates. For example, previous studies found that hand hygiene (HH) compliance before patient contact ranged from 1.1% to 34%, and compliance after patient contact ranged between 27% and 90%.^5,6,10,11^ This variability highlights a critical gap in our understanding of IPC compliance within EMS settings.

Adherence to IPC practices is often impeded by a variety of complex and multifaceted barriers. These include insufficient training, resource limitations, high workloads, and the fast-paced, challenging nature of EMS work.^4,12^ Understanding these obstacles is crucial for developing strategies to improve compliance and ensure the safety of EMS providers and patients.

This scoping review aims to answer the following research questions: What is currently known about EMS providers’ adherence to infection control practices in prehospital settings? What barriers to IPC adherence have been identified in EMS-related research?

## Methods

We conducted this scoping review following the methodological framework described by Arksey and O’Malley.^13^ The framework comprises five stages: identifying the research question; identifying relevant studies; study selection; charting the data; and collating, summarizing and reporting the results. Additionally, we utilized the PRISMA-ScR guidelines wherever applicable during the review.

### Search strategy

We conducted a comprehensive search of databases to identify relevant articles using Embase, PubMed, and CINAHL databases. We also performed a hand search of the reference lists of included articles for any additional relevant studies. We employed a comprehensive search strategy using keywords and synonyms related to EMS, prehospital, infection control guidelines, best practices, protocols, and adherence. Boolean operators (AND, OR) were used to refine the search. We decided to include only studies published from 2010 onward to make sure the review reflects more current infection control practices. This time period aligns with significant revisions in IPC guidelines and the World Health Organization (WHO) introduction of the “Five Moments for Hand Hygiene” in 2009.^14^ By focusing on more recent publications, our goal was to reduce discrepancies and confirm that all included studies relate to standards still in current use today.

### Eligibility criteria

#### Inclusion criteria

- Studies addressing infection control practices amongEMS providers in prehospital settings.
- Studies reporting compliancewith IPC guidelines.
- Studies examining barriers to IPC implementation.
- Published between January 1, 2010, and July 30, 2024
- Published in English.

#### Exclusion criteria

- Studies not focused on EMS providers or prehospital settings.
- Non-English language publications.

### Study selection

Our selection process involved two steps:

1. Title and abstract screening: A systematic screening of titles and abstracts of the results using predefined criteria to ensure their eligibility.
2. Full-text review: Following the initial title and abstract screening, the studies deemed potentially relevant were subjected to a full-text review. This step involved a detailed examination of the studies to confirm eligibility based on inclusion and exclusion criteria.

The screening process was facilitated by Rayyan. Rayyan is a free online tool developed by the Qatar Computing Research Institute, designed to facilitate the screening process for systematic and scoping reviews. This tool enabled reviewers to efficiently manage and categorize studies, facilitating a more systematic and transparent screening process.^15^

### Data extraction and analysis

Data extraction forms were developed to collect relevant information from the included studies. For studies reporting compliance, we reported the following: study details (authors, publication year, study design, and country), compliance rates for the selected practice, and major limitations for each study. For studies exploring barriers, we reported the identified barriers and the number of times they were identified in the studies.

## Results

**Figure 1.**
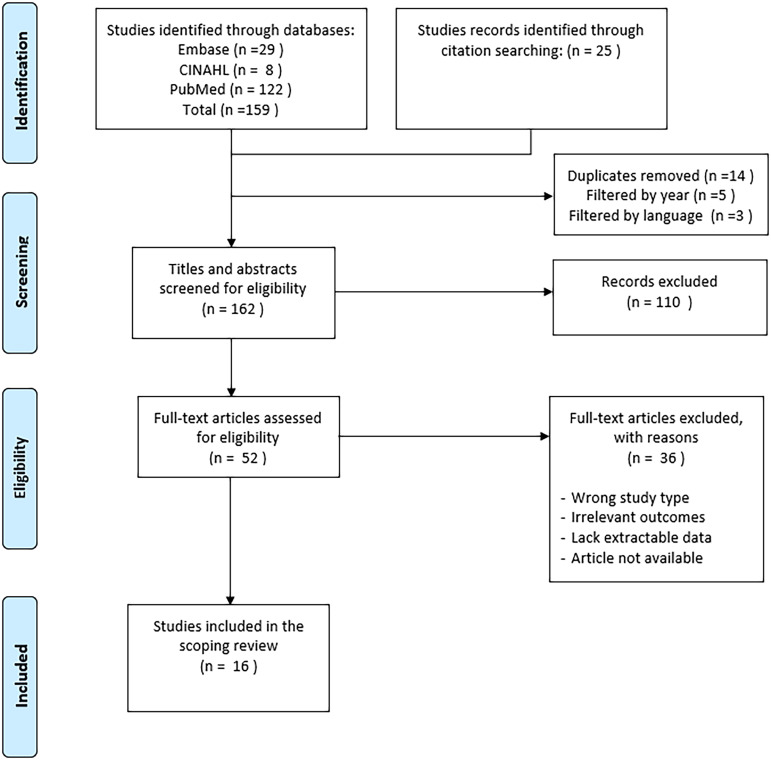
PRISMA flowchart.

The initial search yielded 184 records, 159 from databases, and 25 identified by hand-searching the citation lists of included records. In total, 162 records were identified after applying the following filters: duplicates, non-English, and publication years. After title and abstract screening, 52 studies were selected for full-text review. Of these, 16 studies met the inclusion criteria and were included in the final analysis. The study selection process is summarized in a PRISMA flow diagram (Figure 1).

### Characteristics of included studies

**Figure 2.**
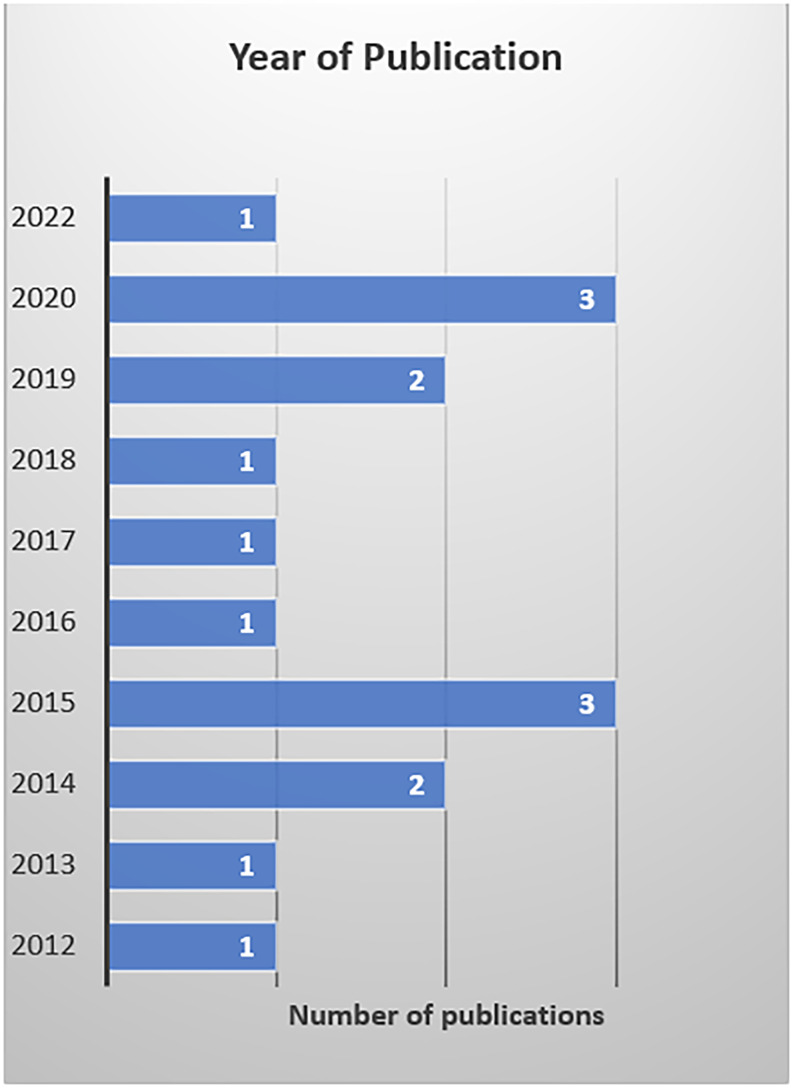
Year of publication.

The included studies involved a range of geographic locations and study designs. The included studies were conducted in the United States 8, Europe 5, Asia 2, and Australia 1. The included studies utilized a mix of quantitative and qualitative methodologies. Based on our findings, all included studies focus on one or two IPC practices, most often gloves and HH. Only six studies used direct observation of EMS providers to assess selected IPC practices. These studies used various methods to record compliance rates. For example, in one study, an EMS team member acted as the observer while another was observed.^16^ In another, observers waited at emergency departments to document EMS teams’ IPC compliance with guidelines.^10^ Among the studies reviewed, only one explored the association between HH compliance and various factors. In that study, the authors conducted univariate and multivariate analyses to determine whether provider level, sex, and glove use were potential factors related to non-compliance with HH using logistic regression. The number of included studies published per year is shown in Figure 2.

### Studies reporting IPC compliance rates

#### Gloves

Our review shows that glove use is among the most investigated IPC practices. As shown in Table 1, eight studies have assessed glove use in prehospital settings. Those studies used different approaches to assess compliance rates. In general, compliance with glove use among EMS providers seems to be higher than other IPC guidelines. The highest compliance rate reported was 100% in a questionnaire-based study.^11^ The lowest compliance rate reported was 52% in a questionnaire-based study.^17^

**Table 1.** Gloves

Study	**Compliance**	**Data collection**	**Limitations**
(Emanuelsson et al., 2013) Sweden(*n* = 68)	69%	Observation – One of the EMS team is the observer, and the other is under observation.	- Limited observation period as the study was conducted over 1 day. - Hawthorne effect is not addressed in the study. - Lack of demographic and contextual details.
(B. E. Bledsoe et al., 2014) USA (*n* = 899)	56.9%.	Observation – EMS teams were observed upon their arrival at a large urban emergency department.	- Lack of on-scene compliance data.- Lack of demographic and contextual details.
(Ho et al., 2014) USA (*n* = 53)	87.6%	Observation	- Lack of demographic and contextual details. - Lack of detailed PPE compliance data.
(Bucher et al., 2015) USA (*n* = 1 494)	“Always” wear gloves 52%	Questionnaire-based	- Risk of social desirability bias, recall bias and selection bias may limit the generalizability of findings.
(Liang et al., 2015) USA (*N* = 516)	Almost always (≥80% of the time) (84.9%)	Questionnaire-based	- Risk of social desirability bias, recall bias and selection bias may limit the generalizability of findings.
(Oh and Uhm, 2016) South Korea (*n* = 907)	68.9%	Questionnaire-based	- Risk of social desirability bias, recall bias and selection bias may limit the generalizability of findings.- Lack of detailed PPE compliance data.- Lack of demographic and contextual details.
(Barr et al., 2017) Australia (*n* = 417)	100%	Questionnaire-based	- Risk of social desirability bias, recall bias and selection bias may limit the generalizability of findings.
(H. S. Vikke, S. Vittinghus, M. Giebner, et al., 2019)Finland, Sweden, Australia and Denmark (*n* = 87)	54%	Observation	- Risk of Hawthorne effect.-Small sample size

#### Hand hygiene

HH is the most commonly investigated practice in prehospital settings; however, previous studies have lacked a standardized method for assessing EMS providers’ compliance with HH. Some studies reported only overall compliance, while others provided more detailed information, including the timing of assessments, often distinguishing between before and after patient contact. Our review shows that only two studies assessed compliance according to the WHO’s Five Moments for Hand Hygiene, as illustrated in Table 2. We used the WHO method due to the lack of standardized assessment methods, which complicates the comparison of findings across studies. Reported compliance rates show notable differences based on the methodology employed in each study. For example, in questionnaire-based studies, compliance rates after patient contact ranged from 67% to 94%. In contrast, direct observation studies reported compliance rates after patient contact between 27% and 72%.

**Table 2.** Hand hygiene

Study	**Compliance**					**Data collection**	**Limitations**
	Before patient contact	Before aseptic procedures.	After risk of body fluids.	After patient contact	After contact with patient surroundings.		
(McGuire-Wolfe et al., 2012) USA (*n* = 228)	NA	NA	NA	94%	NA	Questionnaire-based	- Risk of social desirability bias, recall bias, and selectionbias may limit the generalizability of findings. - Lack of detailed information regarding compliance.
(Emanuelsson et al., 2013)Sweden (*n* = 68)	34%	NA	NA	72%	NA	Observation	In Table 1
(B. E. Bledsoe et al., 2014) USA (*n* = 899)	NA	NA	NA	27.8%	NA	Observation	In Table 1
(Ho et al., 2014) USA (*n* = 258)	1.1%	3.5%	NA	68.9%	NA	Observation	In Table 1
(Jonathan Teter et al., 2015)USA (*n* = 62)	34%	NA	NA	79%	NA	Questionnaire-based	- Risk of social desirability bias, recall bias, and selectionbias may limit the generalizability of findings. - Lack of demographic and contextual details.
(Bucher et al., 2015) USA (*n* = 1 494)	13%	NA	54%	67%	NA	Questionnaire-based	In Table 1
(Liang et al., 2015) USA (*N* = 516)	16.1%*Before gloves use	NA	NA	68.9%*After gloves use	NA	Questionnaire-based	In Table 1
(Barr et al., 2017) Australia (*n* = 417)	32.8%	29.5%	28%	90.4%	28.3%	Questionnaire-based	In Table 1
(H. S. Vikke, S. Vittinghus, M. Betzer, et al., 2019) Denmark (*n* = 457)	–	–	–	–	–	Questionnaire-based* The self-reported HH compliance rate was 90%	Risk of social desirability bias, recall bias, and selectionbias may limit the generalizability of findings.
(H. S. Vikke, S. Vittinghus, M. Giebner, et al., 2019) Finland, Sweden, Australia and Denmark (*n* = 87)	3%	2%	8%	29%	38%	Observation	In Table 1
(Olsson et al., 2022) Sweden (*n* = 214)	–	–	–	–	–	Questionnaire-based* The self-reported HH compliance rate was 85%	Risk of sampling bias from social media recruitment, questionnaire validity issues, and limited generalizability due to overrepresentation of specialist nurses.

#### Masks

This review identified one study that reported on mask use among EMS providers. The available study indicated that EMS providers were more likely to use masks when transporting patients suspected of having an infectious respiratory condition. Table 3 summarizes the findings of the included study.

**Table 3.** Masks

Study	**Compliance**	**Data collection**	**Limitations**
(Oh and Uhm, 2016) South Korea (*n* = 907)	- Overall 24.7% - 93.8% When transferring patients with respiratory symptoms - 86.0% When transferring trauma patients with bleeding.	Questionnaire-based	In Table 1

#### Eye Protection/Goggles and Gowns

Two studies have assessed EMS providers’ compliance with eye protection/goggles and gowns. These studies are shown in Table 4.

**Table 4.** Eye protection/goggles and gowns

Study	**Compliance**			**Data collection**	**Limitations**
(Emanuelsson et al., 2013) Sweden (*n* = 68)	The use of gowns was correct in 91%			Observation –	In Table 1
(Oh and Uhm, 2016) South Korea (*n* = 907)		Goggle	Gowns	Questionnaire-based	In Table 1
	Overall When transferring patients withrespiratory symptoms. When transferring trauma patients with bleeding.	1.7% 25.5% 38%	.4% 11.5% 23.1%		

#### Surface disinfection

Surface disinfection refers to the cleaning of ambulance interiors after a call. Our review identified four studies that examined EMS providers’ compliance with this IPC practice. Since this procedure is typically performed at hospitals after the conclusion of a case, it is more feasible to use direct observation to record compliance. Table 5 presents the studies that reported on this practice.

**Table 5.** Surface disinfection

Study	**Compliance**	**Data collection**	**Limitations**
(B. E. Bledsoe et al., 2014)USA (*n* = 100)	33.3%.	Observation –	In Table 1
(Liang et al., 2015)	-At the beginning of shift 52.1%. -At some point during shift 58.9%.-After patient encounter 14.1%. -After patient encounters resulting in visible contamination of the environment 53.8%.	Questionnaire-based	In Table 1
(Vikke et al., 2018) Denmark (*n* = 80)	MC was registered in all 80 (100%) ambulances.	Observation –According to Danish prehospitalhygiene guidelines, Moderate cleaning (MC) must be conducted after every patient course.	In Table 1
(Ventura et al., 2020) USA (*n* = 192)	53% of providers reported decontaminating the patient compartment of their EMS unit after each patient contact	Questionnaire-based	- Risk of social desirability bias, recall bias and selectionbias may limit the generalizability of findings. - Lack of detailed PPE compliance data. - Lack of demographic and contextual details.

#### Equipment disinfection

The disinfection of reusable equipment is a critical component of IPC practices in prehospital settings. Despite its importance, only three studies in our review specifically evaluated EMS providers’ compliance with the disinfection of reusable equipment. In our review, we sought to include studies that provided data on the disinfection of the items shown in Table 6: stretcher, backboard, blood pressure cuff, stethoscope, cervical collars, splints, ECG monitor/ defibrillator, ECG monitor/defibrillator cables, pulse oximeter, suction unit, and LUCAS device.

**Table 6.** Equipment disinfection

Study	**Compliance**	**Data collection**	**Limitations**
(B. E. Bledsoe et al., 2014) USA (*n* = 100)	Stretcher (55%) Backboard (31.6%) ECG monitor (33.8%) ECG cables (36.5%) Stethoscope (4.1%)	Observation –	In Table 1
(Liang et al., 2015)	At the beginning of the shift (47.9%) During each shift (41.2%) After patient encounter (85.9%) After patient encounters resulting in visible contamination (46.3)	Questionnaire-basedEquipment/stretcher disinfection	In Table 1
(Ventura et al., 2020)USA (*n* = 192)	Stethoscope (47%) after each patient contact	Questionnaire-based	In Table 5

#### Barriers

We identified barriers to IPC compliance among EMS providers from the reviewed articles, which reported these barriers either directly or indirectly. Some studies identified barriers directly by clearly asking EMS providers about the factors that hinder their compliance, or by observing their behavior and practices in real-time. Other studies did not specifically identify barriers but offered findings that can be interpreted as barriers. For example, when a study explored measures considered effective to improve IPC compliance and found that providers emphasized the importance of consistent personal protective equipment (PPE) availability, this implies that inadequate PPE supply is perceived as a barrier. Table 7 presents how often each barrier was reported in the studies we reviewed.

**Table 7.** Frequency of reported barriers

**Barriers**	**Number of Studies**	**Barriers**	**Number of Studies**
Operational pressure and time constraints	7	Insufficient training/education:	6
Fatigue	1	No need to use PPE with “low-risk” patients	7
Concerns about patient comfort	1	PPE interfere with the ability to do procedures:	5
Skin reactions to PPE items	1	Forgetfulness	5
Insufficient organizational support	3	PPE may not be readily available/ Insufficient PPE supply	9
Unpredictable work environment	1	Inadequate PPE fit	2

## Discussion

Overall, the findings of this review show notable inconsistencies in IPC compliance rates among EMS providers in prehospital settings. Also, this review identified a wide range of barriers to IPC compliance among EMS providers.

### Compliance rates across IPC practices

No research to date has employed a comprehensive, direct-observation approach to explore EMS providers’ adherence to all relevant IPC practices.

### Gloves

Half of the included studies that reported mask compliance relied on self-reported data from questionnaires, while the other half used direct observation methods, applying different approaches to monitor compliance. For example, in one study, observations were made upon the arrival of EMS units to the emergency department, so no actual data of EMS provider compliance during field operations.^10^ In another study, two external observers monitored the EMS teams, which meant that the providers were aware they were being observed, even though they were not informed of the focus of observations.^23^ Such an approach could have influenced their behavior, a phenomenon known as “Hawthorne effect”.

### Hand hygiene

Practicing efficient HH is vital in the EMS context, especially since EMS providers often operate in uncontrolled environments and many procedures are becoming invasive.^23^ In one study, the investigators cultured EMS providers’ hands and found that 77% carried heavy bacterial loads following patient care.^6^ In this review, we observed that HH compliance among EMS providers is remarkably low, with rates as low as 1% before patient contact. Generally, EMS providers are more likely to practice HH after patient contact compared to before.

### Masks

The difference in compliance rates for mask usage while caring for patients with infectious disease symptoms compared to asymptomatic patients may indicate a gap in awareness or perceived risk among many EMS providers. Since the included study was conducted before the COVID-19 pandemic, it would be interesting for future research to assess the impact of COVID-19 on EMS providers’ behavior, especially given the heated debate that followed it.

### Eye protection/goggles and gowns

In this review, the studies included show that EMS providers have not always consistently used eye protection and gowns as part of standard precautions. Similar to masks, the adherence of EMS providers to these practices was clearly affected by the presence of infectious disease symptoms in patients.

### Surface disinfection

Surface disinfectionis significant in controlling the risk of MTAI. Previous research shows that ambulances are usually contaminated with different types of pathogens. Accordingtoa recent systematic literature review that explored microbial contamination on ambulance surfaces, the majority of surfaces analyzed in the studies included in the review were contaminatedwith organisms commonly associated with HAIs, highlighting a significant issue in this area.^26^ Although monitoring this procedure should be easier for investigators, as it is typically conducted in hospitals, only four studies have explored this area.

### Equipment disinfection

The standard procedure for cleaning equipment involves disinfecting all reusable items that might have come into contact with the patient or been exposed to contamination during the call. In their 2022 review, Obenza et al found that most EMS equipment are contaminated. In our review, only three studies explored EMS providers’ compliance with equipment cleaning. The stretcher was the most commonly disinfected item, with disinfection performed 55% of the time. No other reusable equipment exceeded 50%. Incomplete disinfection poses an elevated risk of transmitting infections to patients or EMS providers.

### Barriers to IPC compliance

We classified barriers into three primary categories: individual, organizational, and operational. Our classification scheme represents a novel contribution to literature. While previous studies have identified different barriers, no standardized framework was available to categorize them systematically. By developing this classification, we provide a structured approach to interpreting and comparing findings across studies, potentially guiding future research and interventions.

#### Individual

No need to use PPE with “low-risk” patients,forgetfulness, fatigue, skin reactions to PPE items, concerns about patient comfort.

#### Organizational

Insufficient training/education, PPE may not be readily available/Insufficient supply, insufficient support.

#### Operational

PPE interferes with the ability to do procedures, inadequate PPE fit, operational pressure and time constraints, unpredictable work environment.

### Individual

The most common individual barrier was the perception that PPE is not needed when caring for patients who are considered low-risk patients. This barrier highlights an obvious knowledge gap among EMS providers regarding the potential transmission risks associated with asymptomatic individuals. The next most reported individual barrier was forgetfulness. The fast-paced and high-stress nature of EMS work could lead EMS providers to overlook some protocols, such as HH.

### Organizational

Organizational barriers were the most frequently cited type of barrier, indicating that structural and systemic issues within EMS agencies significantly affect compliance with IPC guidelines. The most common organizational barrier was insufficient availability of PPE. Resource limitation for some equipment was particularly challenging during COVID-19. In a survey of EMS providers conducted during COVID-19, only 48% reported having access to N95 masks when needed.^25^ Additionally, training and education were identified as critical barriers. Six studies highlighted insufficient training as a significant factor contributing to suboptimal compliance. Inadequate training or lack of IPC updates may leave providers unaware of the latest guidelines or unprepared to implement them when needed. Organizational support, including educational programs, leadership engagement, and clear communication, is crucial for fostering a culture of safety in prehospital settings. Insufficient organizational support may result in providers deprioritizing IPC practices under operational demands.

### Operational

Operational barriers were primarily related to the nature of EMS work itself. EMS providers often operate in unusual environments and sometimes under extreme pressure, with limited time to effectively follow PPE.^23,27^ Furthermore, certain PPE, such as gloves or goggles, was reported to interfere with clinical procedures or the ability to communicate with patients, leading providers to skip these protective measures in critical situations.^19,28^ Operational barriers might be the hardest set of barriers to overcome, as they are often influenced by difficult-to-control factors. Mitigating these barriers requires a careful balance between ensuring providers’ and patients’ safety and maintaining the quality of emergency care.

### Implications for policy

The findings of this review have significant implications for both practice and policy. First, EMS agencies should prioritize the development and implementation of comprehensive, evidence-based training programs that emphasize the importance of IPC practices and provide practical training on how to follow these practices in the prehospital environment, as highlighted by some studies cited in this review.^5,17^ Training programs should be regular, scenario-based, and tailored to address specific knowledge gaps and barriers identified by EMS providers, such as the perceived belief that PPE are unnecessary in low-risk situations and the challenges associated with operational pressures.

Second, EMS agencies should adopt mechanisms that could help monitor compliance and identify areas for improvement. Several studies included in this review reported considerable non-compliance with some IPC practices, with compliance rates reported as low as 1% in some instances.^5^ Regular audits and feedback could serve as critical tools for strengthening adherence to ICP protocols. In a Swedish study, ambulance staff were asked which interventions could improve HH compliance, and over 80% of respondents suggested that receiving feedback would be an effective method.^23^ Such efforts can boost compliance and establish a proactive safety mindset, ultimately contributing to the EMS system’s capacity and overall efficiency.

Finally, addressing resource limitations and ensuring adequate organizational support are essential for improving IPC compliance. Of the barriers identified in this review, resource limitations were consistently noted as the biggest challenge faced by EMS providers, highlighting the critical need for adequate support and resources to facilitate effective infection control practices. EMS agencies should work with policymakers and stakeholders to secure funding and to establish mechanisms that ensure a continuous supply of IPC equipment. Adequate PPE sourcingand stockpiling strategies should be implemented to prevent shortages, especially during high-demand periods, such as COVID-19.

### Strengths and limitations

This scoping review has some limitations that should be considered when interpreting our findings. The inclusion of only English-language studies may have led to the exclusion of relevant research published in other languages, potentially introducing a language bias. The variability in study designs and methodologies, particularly in measuring IPC compliance, may limit the generalizability and reliability of the findings. Additionally, most of the included studies relied on self-reported data, which may be subject to social desirability bias, recall bias, and response bias, potentially leading to an overestimation of compliance rates and limiting the generalizability of the findings.

Despite these limitations, to the best of our knowledge, this review is the first to provide a detailed overview of the published literature on IPC compliance among EMS providers, offering in-depth data on adherence across different IPC practices. A key strength of this review is the comprehensive search strategy that involved multiple databasesand the inclusion of a hand-search of reference lists to capture relevant studies. This approach minimized the risk of missing relevant studies and ensured a thorough mapping of the existing evidence on IPC compliance and barriers among EMS providers. In addition to highlighting the factors and barriers influencing EMS providers’ IPC adherence, this review gathers data from different parts of the world, offering a global overview of IPC practices within prehospital care settings.

### Directions for future research

The findings of this review reveal that many key areas need additional research to enhance our knowledge of IPC adherence among EMS providers in prehospital environments. There is a pressing need for observational studies that assess real-time IPC compliance among EMS providers. Most existing studies relied on self-reported data, which, as indicated earlier, are subject to social desirability, recall, and response biases, often resulting in an overestimation of compliance rates.^29–31^ In one of the included studies, researchers identified a noticeable difference in EMS providers’ responses when asked about their own compliance with HH compared to that of their colleagues. Most respondents, 94%, reported consistently practicing HH after patient transport, but only 71.9% felt that their colleagues followed the same practices after patient contact. This difference is a clear example of the social desirability bias.^21^ By capturing real-time data on EMS providers’ practices, such studies would offer valuable insights into actual compliance behavior, thus filling a critical gap in the current literature. Additionally, many of the reviewed studies were conducted in the preCOVID era. The pandemic has influenced IPC practices, awareness, and compliance in healthcare settings, including among EMS providers.^32^ Therefore, there is a need for more recent studies that evaluate IPC compliance in the postpandemic context. Such research would help assess whether any changes implemented during the pandemic have translated into long-term changes among EMS providers. Furthermore, existing studies tend to focus on one or two IPC practices, without evaluating other IPC practices simultaneously. There is currently no single study that provides a detailed assessment of EMS providers’ adherence to a complete set of IPC guidelines.

Future research on barriers to compliance should employ an innovative approach, such as a mixed-methods approach, to get robust data. The mixed-methods approach integrates both quantitative and qualitative data to offer a more comprehensive understanding of addressed issues.^33^ Given the unpredictable nature of prehospital settings, mixed-methods design can be particularly beneficial in EMS research.^34,35^ This approach is particularly critical for understanding barriers to IPC adherence, as these obstacles are often complex and multifaceted. This approach combines both data types, providing a richer understanding that neither method can capture alone. In conclusion, addressing these research gaps through well-designed studies, such as observational and mixed methods approaches, will provide reliable results of IPC compliance and barriers faced by EMS providers. Addressing these gaps is crucial for developing targeted interventions and policies that can enhance compliance, ultimately improving the quality of EMS.

## Data Availability

Not applicable.
